# Development of environment-responsive self-propelling robots with shape-preserving exoskeletons for directed interfacial motion

**DOI:** 10.1039/d6ra03989h

**Published:** 2026-08-25

**Authors:** Ittetsu Toda, Hideyuki Sawada

**Affiliations:** a Department of Pure and Applied Physics, Graduate School of Advanced Science and Engineering, Waseda University 3-4-1 Okubo Shinjuku-Ku Tokyo 169-8555 Japan i-toda@toki.waseda.jp; b Faculty of Science and Engineering, Waseda University 3-4-1 Okubo Shinjuku-Ku Tokyo 169-8555 Japan

## Abstract

Autonomous soft robots capable of converting chemical energy into mechanical motion possess significant potential for applications such as targeted transport and environmental monitoring. In particular, systems that autonomously modulate their behavior in response to environmental stimuli are essential for practical implementation. Self-propelling droplets driven by the Marangoni effect are representative of chemical propulsion; however, their trajectory is inherently stochastic due to the random formation of interfacial tension gradients, making directional control difficult. Thus, establishing stable rectilinear motion remains a critical challenge. In this study, we have developed a pH-responsive gel robot powered by self-propelling droplets and propose a design strategy based on structural constraints to enhance directional controllability. Specifically, 1-pentanol-containing poly(*N*-isopropylacrylamide-*co*-acrylic acid) (PNIPAm-*co*-AAc) gels are synthesized and evaluated in a pH 12 solution. At high pH, deprotonation of the acrylic acid carboxyl groups induces significant swelling through electrostatic repulsion within the polymer network. This pH-dependent expansion triggers the release and diffusion of internal 1-pentanol into the surrounding aqueous phase, creating interfacial tension gradients that induce Marangoni convection. While unconstrained gels exhibit random motion, the introduction of a 3D-printed resin exoskeleton geometrically restricts the reaction interface and localizes the convection formation. Particle Image Velocimetry (PIV) confirms that the convection field is aligned along the robot's central axis; thereby, stable straight-line propulsion was achieved. These results demonstrate that integrating pH-responsive swelling with mechanical constraints provides a robust design principle for programming the motion of chemically driven soft robots.

## Abstract 2

A pH-responsive soft robot driven by 1-pentanol Marangoni convection achieves autonomous, directional interfacial motion using a shape-preserving 3D-printed exoskeleton.

## Graphical abstract

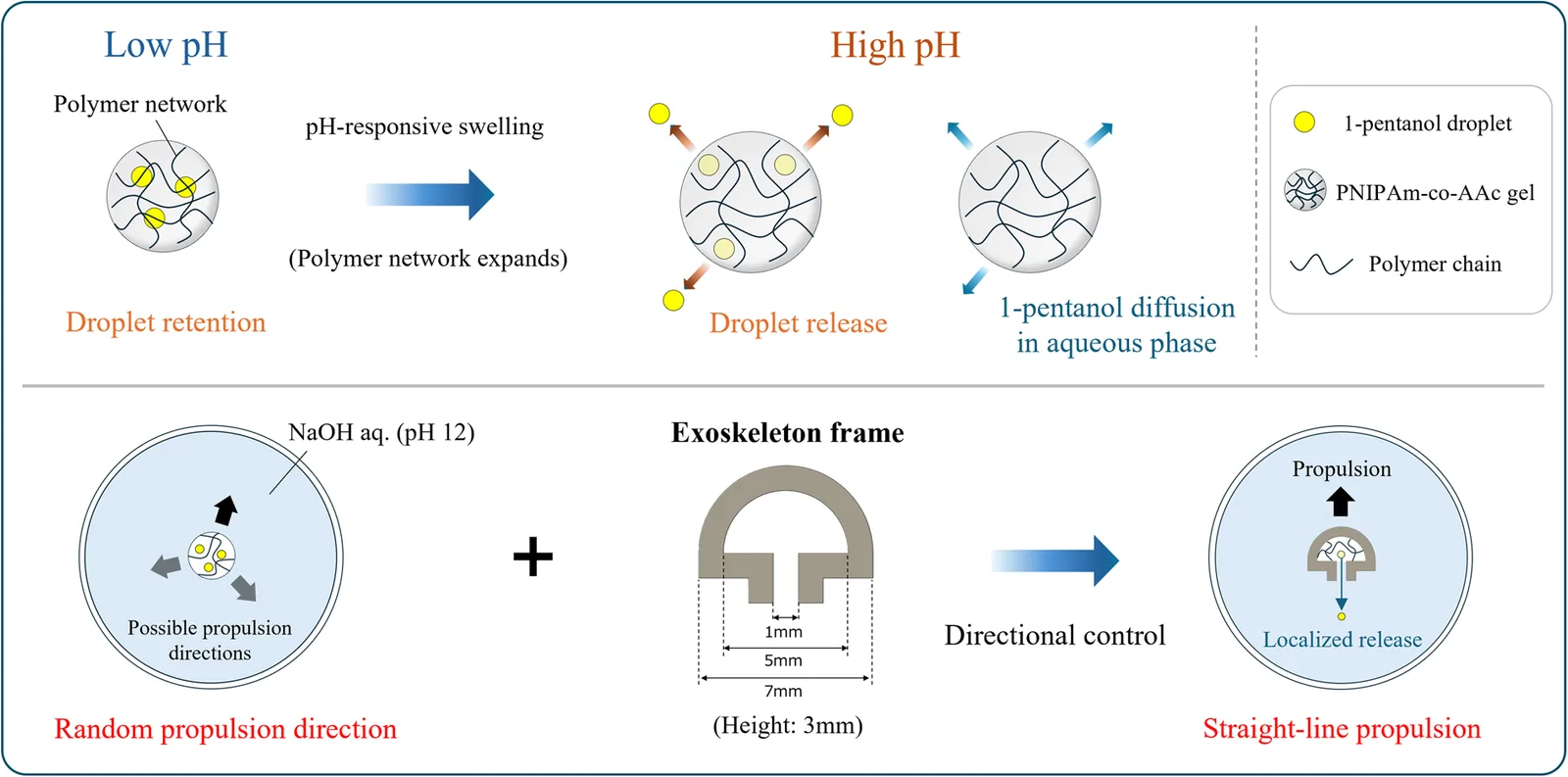

## 1 Introduction

In recent years, autonomous robots that rely solely on energy derived from chemical reactions and molecular diffusion without external power supplies or mechanical manipulation have attracted significant attention.^1–6^ This research is motivated by the biomimetic engineering of efficient chemical-to-mechanical energy conversion mechanisms found in living organisms. By applying these mechanisms, such systems are expected to find applications in soft robotics and the medical field.

Self-propelled droplets are a representative example of autonomous motion in solution. This phenomenon occurs when a droplet, placed in a solution, exhibits spontaneous movement due to convection induced by the Marangoni effect, which originates from gradients in interfacial tension on the droplet surface.^7–17^ While this mechanism is expected to serve as a power source for robots that do not require external energy, precise control over the direction and velocity remains a challenge because the formation of the interfacial tension gradient is stochastic.^18,19^

To address this challenge, we have attempted to establish directional control methods using exoskeletons.^20,21^ However, since these conventional systems propel continuously, it is difficult to autonomously switch their motion states in response to the changes of the external environment. Therefore, this study aims to construct a novel self-propelled system that integrates directional control with environment-responsive, autonomous motion switching.

Specifically, this study proposes a composite system that integrates stimuli-responsive hydrogels with self-propelled droplets.

Stimuli-responsive hydrogels are functional polymers that exhibit swelling and shrinking behavior in response to external triggers such as temperature, pH,^22^ or light. By encapsulating self-propelled droplets within these stimuli-responsive hydrogels, the gel functions as a control unit for the droplets. Specifically, it is expected that the site and volume of droplet release can be spatially and temporally controlled in response to external stimuli. This allows for the intentional manipulation of convection patterns around the hydrogel, leading to the development of environment-responsive autonomous systems. Therefore, this study aims to construct a gel robot with encapsulated self-propelled droplets and investigate the controllability of its self-propelled behavior in response to environmental changes. This autonomous motion control technology, based on environmental responsiveness, is expected to find applications in drug delivery systems (DDS)^23–28^ that sense the chemical environment of specific areas to deliver drugs precisely.

Self-propelled droplets exhibit spontaneous motion by forming convective flows driven by the Marangoni effect. This effect occurs due to the non-uniformity of interfacial tension on the droplet surface, which causes fluid to flow from regions of lower interfacial tension toward regions of higher tension. Below, the specific driving principle is described using 1-pentanol, the substance employed in this study, as an example.

1-Pentanol is partially soluble in water. When it is dropped into an aqueous phase, a concentration gradient is formed on the droplet surface, leading to an interfacial tension gradient. Regarding its self-propelled behavior, it has been reported that droplets with volumes between 0.1 and 200 µL deform into a boomerang-like shape and move in the convex direction.^29^ This behavior is explained by the convective flows formed both inside and outside the droplet.

Suzuki *et al.* analyzed the internal and external convection formed in self-propelled 1-pentanol droplets.^21^

First, the convection within the droplet is attributed to the curvature of its shape. Specifically, the difference in curvature between the front and rear of the droplet causes a variation in the diffusion rate of 1-pentanol, leading to a local concentration gradient. Since this concentration gradient is directly linked to the interfacial tension gradient, it induces internal convection that flows from regions of lower to higher interfacial tension. This convection functions as an internal driving force.

Next, the external convection surrounding the droplet is primarily caused by the diffusion of 1-pentanol into the aqueous phase. Since 1-pentanol is less dense than water, convection resulting from that diffusion occurs near the upper surface of the solution, flowing away from the droplet and then returning in a loop. When the droplet shape is asymmetric, the diffusion rate increases on the side with the larger contact area, leading to the formation of stronger convection. In other words, the external driving force is the surrounding convection generated by diffusion; the relatively high velocity of this convection pushes and propels the droplet.

In summary, the self-propulsion of 1-pentanol droplets is achieved through the simultaneous action of internal convection driven by interfacial tension gradients and external convection driven by diffusion, resulting in sustained self-propulsion.

## 2 Materials

*N*-Isopropylacrylamide (NIPAm), acrylic acid (AAc), *N*,*N*′-methylenebisacrylamide (MBAA), ammonium persulfate (APS), *N*,*N*,*N*′,*N*′-tetramethylethylenediamine (TEMED), and 2-hydroxy-2-methylpropiophenone (Tokyo Chemical Industry Co., Ltd), 1-pentanol, phosphate-buffered saline (PBS), sodium hydroxide (NaOH), and boron nitride (FUJIFILM Wako Pure Chemical Corporation), Tween 80 (Bio-Medical Science Co., Ltd), Lumisis Marker (Central Techno Co., Ltd), citric acid (Kenei Pharmaceutical Co., Ltd) are prepared for the experimental system. All chemicals were used as received without further purification. Deionized water was used in all experiments.

## 3 Preliminary experiment

### 3.1 Experimental methods

**Scheme 1:**
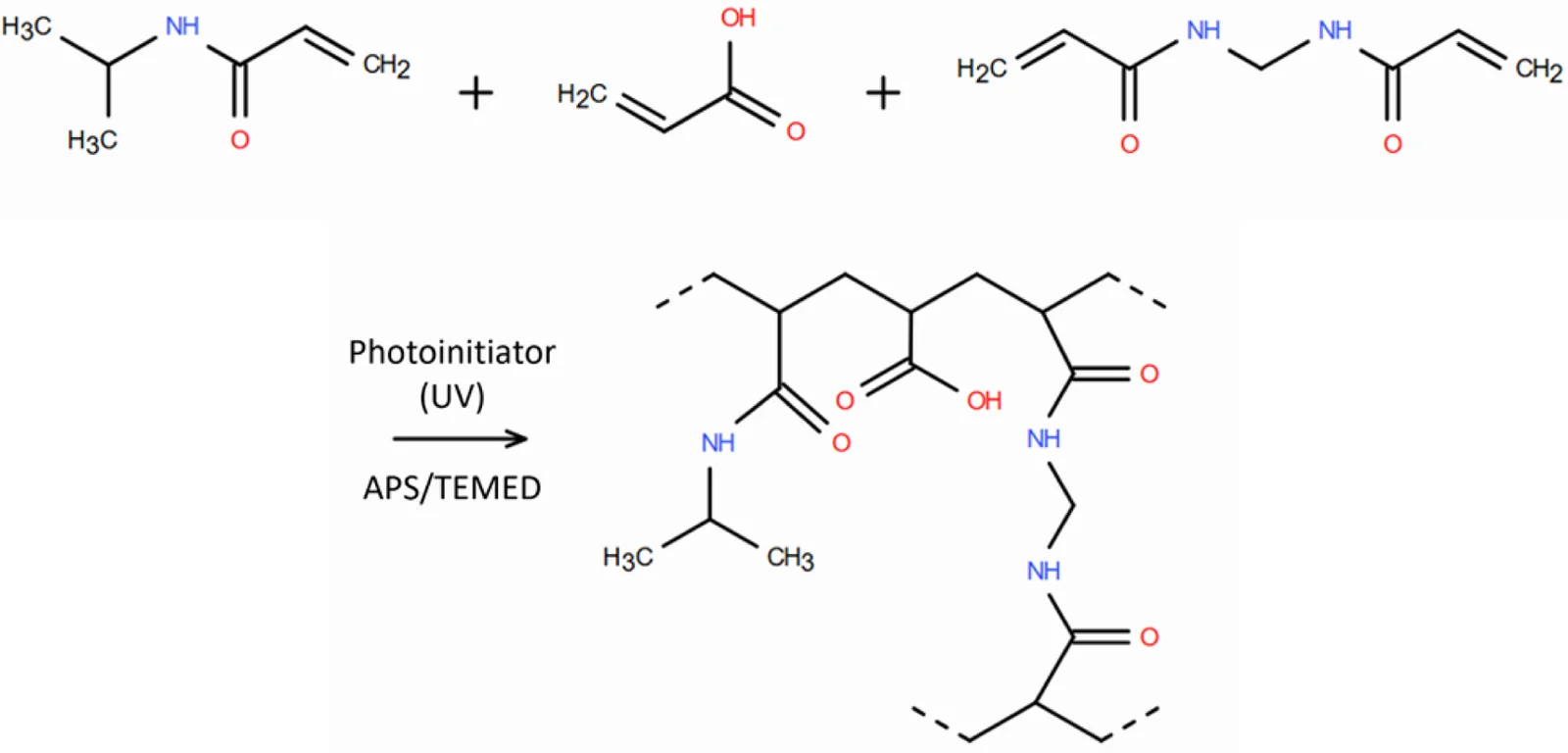
Synthetic route of the PNIPAm-*co*-AAc hydrogel.

In this study, gel robots with encapsulated self-propelled droplets are constructed using three different methods, and their environment-responsive autonomous behavior is evaluated. The PNIPAm-*co*-AAc hydrogel is synthesized *via* radical polymerization. The corresponding reaction is illustrated in Scheme 1.

**Fig. 1:**
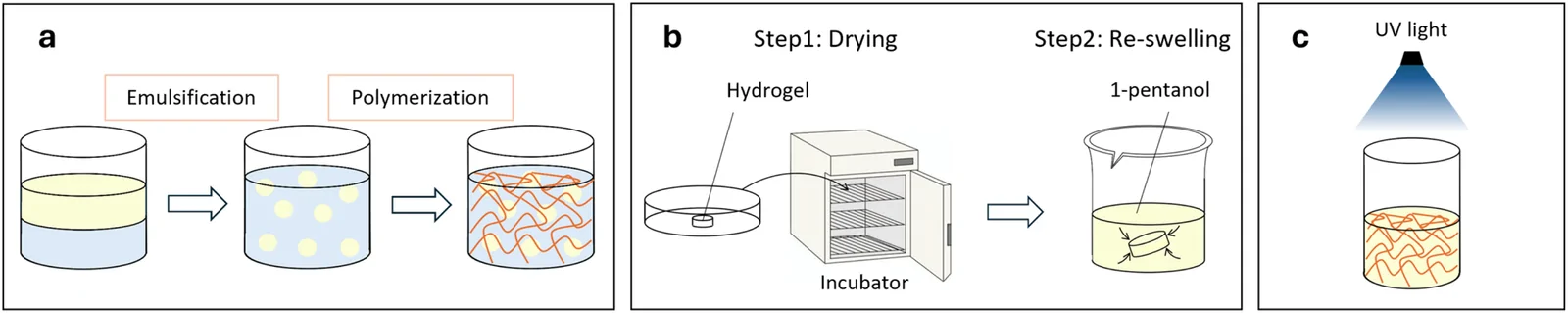
Schematic illustration of the three fabrication methods. ((a): Method 1, (b): Method 2, (c): Method 3).

First, for Method 1, the emulsion-templated gelation method proposed by Tokuyama *et al.*^30^ is adopted. An emulsion gel is a material characterized by a structure where numerous micro-oil droplets are dispersed and immobilized within a gel network. This is achieved by adding an oil phase to a gel precursor solution to create an emulsion before polymerization. A schematic diagram of the emulsion gelation method is shown in Fig. 1(a).

In our system, 1-pentanol is used as the oil phase. By employing the emulsion-templated gelation method, we fabricate gel robots with self-propelled droplets dispersed throughout the polymer network. When the resulting gel robot is immersed in a basic solution, it is hypothesized that the gel will swell and release the micro-oil droplets. This release is expected to induce convection in the surrounding environment, thereby propelling the gel robot.

We prepare the hydrogel robots by synthesizing an emulsion consisting of aqueous and oil phases. The aqueous phase is prepared by dissolving 282 mg of NIPAm, 34 µL of AAc, and 10 mg of MBAA in 2.6 mL of deionized water. Separately, the oil phase is prepared by mixing 1500 µL of 1-pentanol and 0.4 g of Tween 80. We combine these phases and emulsified the mixture using a magnetic stirrer (RS-6AN, AS ONE) for 15 minutes. To initiate polymerization, 10 mg of APS dissolved in 0.4 mL of deionized water is added to the precursor solution along with 20 µL of TEMED. The mixture is stirred for 15 minutes, then sealed in a vial and maintained at a constant temperature of 25 °C for 24 hours to ensure complete polymerization.

**Fig. 2:**
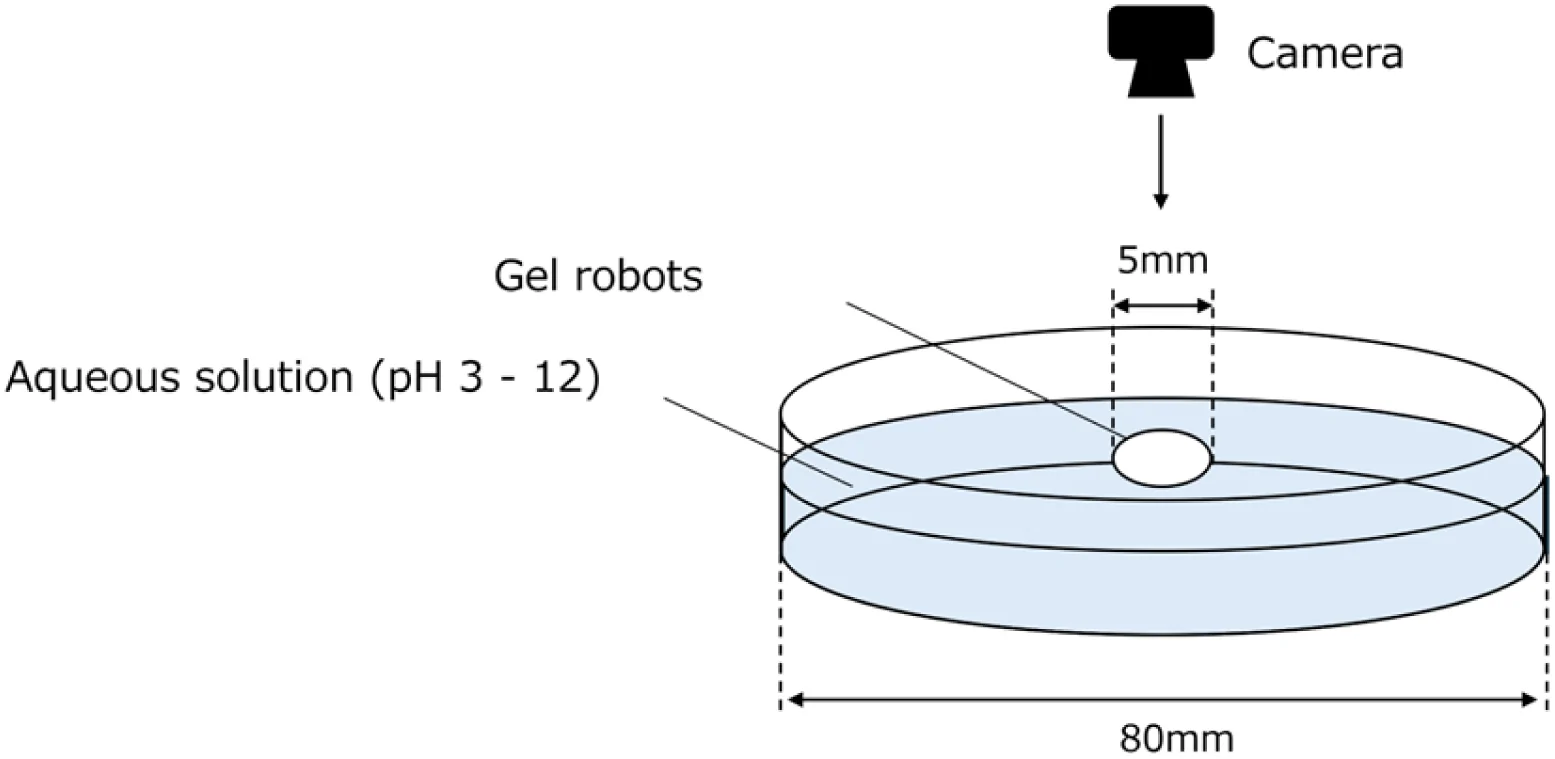
Schematic illustration of the experimental setup.

The resulting hydrogel is cut into disk-shaped samples with a diameter of 5 mm. As a preliminary experiment to evaluate the environmental responsiveness, aqueous solutions adjusted to various pH levels (pH 3, 6, 9, and 12) are prepared using NaOH and citric acid. We pour 30 mL of each solution into a Petri dish with a diameter of 8 cm. Before immersion, each hydrogel disk is washed with PBS and carefully patted dry. We then record the motion of the hydrogel using a video camera (EOS 80D, Canon Inc.). During the observation, when a gel robot came into contact with the Petri dish wall and stopped moving, it is repositioned to the center of the dish to continue the motion recording. The experimental setup is shown in Fig. 2. Finally, the self-propulsion behavior is analyzed using video analysis software, Kinovea. All weighing procedures are conducted using an analytical balance (GH-252, A&D Company, Ltd).

Method 2 involves the fabrication of a porous organogel robot utilizing a drying and re-swelling process. In this approach, a hydrogel is first synthesized in an oil-free aqueous system. By drying the hydrogel to remove internal water molecules, a microporous structure is formed. Subsequently, the resulting gel is immersed in 1-pentanol to allow for re-swelling.

The experimental setup is shown in Fig. 1(b). This process successfully produces a porous organogel that retains droplets within its internal structure.

The experimental procedure is described as follows. The apparatus for mixing and weighing are consistent with those described in Method 1. Photopolymerization is conducted using a desktop UV curing system (Handy 100, HLR100T-2, AS ONE Corporation) and a high-pressure mercury lamp power supply (HB100A-1, AS ONE Corporation). Drying is performed using an incubator (DNE600, Yamato Scientific Co., Ltd).

To construct the porous organogel robot, we first prepare a precursor solution by adding 282 mg of NIPAm, 34 µL of AAc, and 11 mg of MBAA to 2 mL of deionized water. This mixture is stirred for 15 minutes using a magnetic stirrer to ensure complete dissolution. We then add 4 µL of 2-hydroxy-2-methylpropiophenone as a photoinitiator and stir the solution for an additional 30 seconds. The solution is subsequently irradiated with UV light from above for 30 minutes to complete the photopolymerization.

Following the synthesis, the resulting hydrogel is placed in an incubator at room temperature and allowed to dehydrate for 12 hours. The resulting dried gel is immersed in 1-pentanol for 12 hours, during which the polymer network re-swells as it incorporates the solvent.

The self-propulsion behavior and convection patterns of the prepared gel are analyzed using the same experimental and analytical procedures as described in Method 1.

Method 3 involves the fabrication of a 1-pentanol solvent-based organogel robot. In this approach, the solvent used in gel synthesis is changed from water to 1-pentanol. The objective is to shorten the fabrication time and impart a high-concentration droplet retention capacity to the gel.

We first formulate an organogel precursor solution by adding 282 mg of NIPAm, 34 µL of AAc, 11 mg of MBAA, and 8 mg of Tween 80 to 2 mL of 1-pentanol. This mixture is stirred for 30 minutes using a magnetic stirrer to complete dissolution. Next, we add 4 µL of 2-hydroxy-2-methylpropiophenone as a photoinitiator to the solution and stir it for an additional 30 seconds. To initiate the polymerization process, as shown in Fig. 1(c), the mixture is irradiated with UV light from above for 30 minutes. Following the completion of photopolymerization, the self-propulsion behavior of the resulting organogel is evaluated according to the same experimental and analytical procedures established in Method 1. The equipment used for mixing, weighing, and photopolymerization is identical to that used in Methods 1 and 2.

### 3.2 Results and discussion

**Fig. 3:**
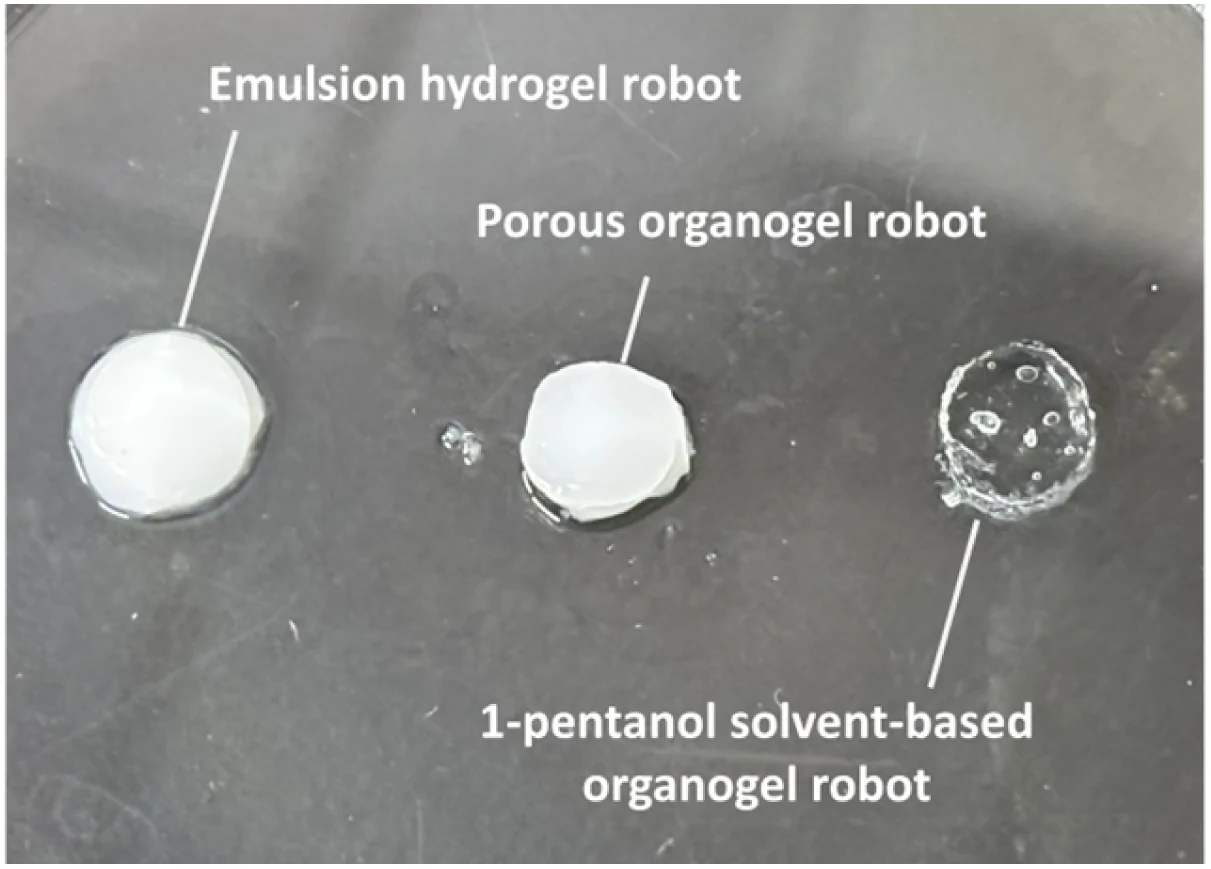
The gel robots prepared by the three different methods.

**Fig. 4:**
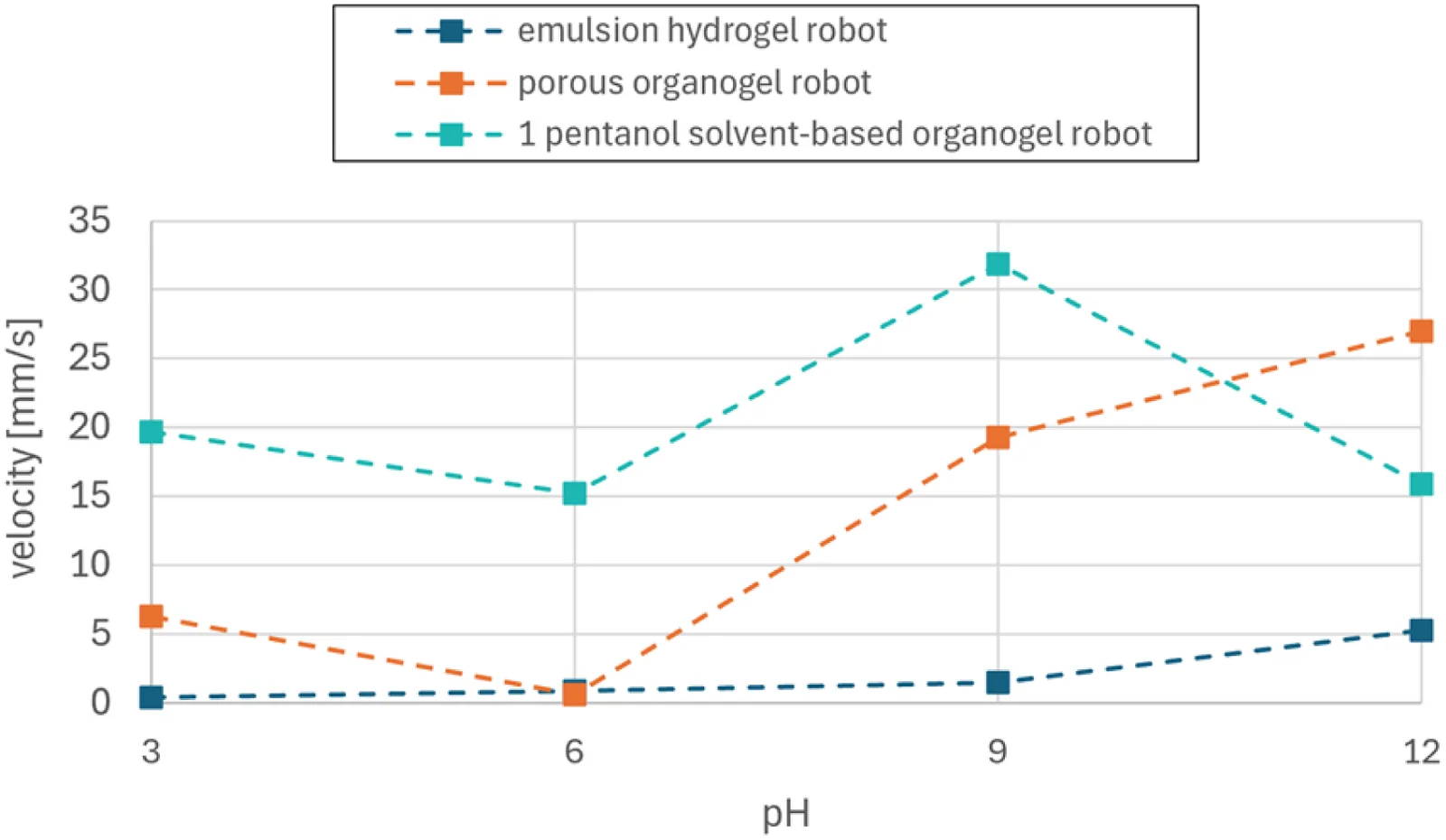
Comparison of the maximum velocities of the gel robots prepared by three different methods under various pH conditions.

The gel robots prepared by using the three different methods are shown in Fig. 3. These robots were immersed in aqueous solutions at pH 3, 6, 9, and 12, respectively, and their propulsion behavior was recorded by using a video camera. Their respective maximum velocities are calculated, which are presented in Fig. 4. To exclude the initial random behavior when a gel robot is released in immersed solution, data recording started when the propulsion become stable. As the robots approached the wall of the Petri dish, the propulsion data were also excluded from the analysis.

**Fig. 5:**
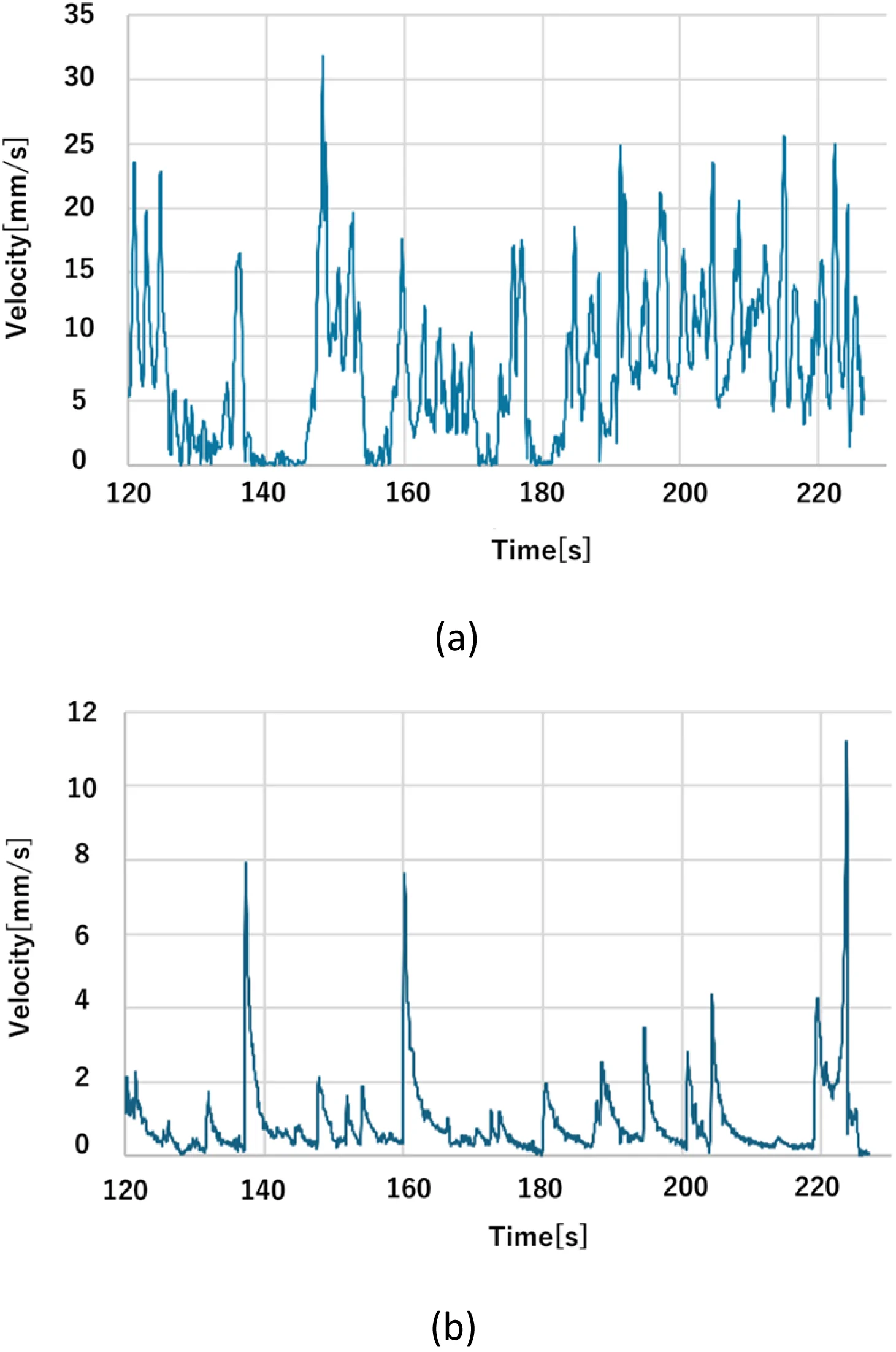
Velocity–time (*v*–*t*) profile of the 1-pentanol solvent-based organogel robot. (a) In pH 9 aqueous solution. (b) In pH 12 aqueous solution.

As found in Fig. 4, the maximum propulsion velocity of the three gel robots is highly dependent on the environmental pH. Particularly in the cases of the emulsion hydrogel robot and the porous organogel robot, the maximum velocity increases as the conditions become more basic. This trend suggests that the dissociation of carboxyl groups increases the volume of released droplets. In the case of a 1-pentanol solvent-based organogel, the velocity–time (*v*–*t*) graph reveals a clear difference. The gel maintains continuous and erratic propulsion at pH 9 (Fig. 5a). In contrast, at pH 12, it exhibits pulsatile propulsion with distinct velocity peaks and resting periods (Fig. 5b). Analytically, this pulsatile behavior makes it much easier to quantify the motion because each propulsion event is clearly separated. Therefore, we selected pH 12 as the optimal condition to investigate the detailed propulsion behavior of the introduced gel robots. Consequently, all subsequent analyses of the self-propulsion behavior and the introduction of directional control methods are consistently conducted under the pH 12 condition.

## 4 Proposed method

### 4.1 Evaluation of self-propelled behavior

Based on the results of the previous section, the self-propelled behavior under pH 12 conditions is evaluated.

**Fig. 6:**
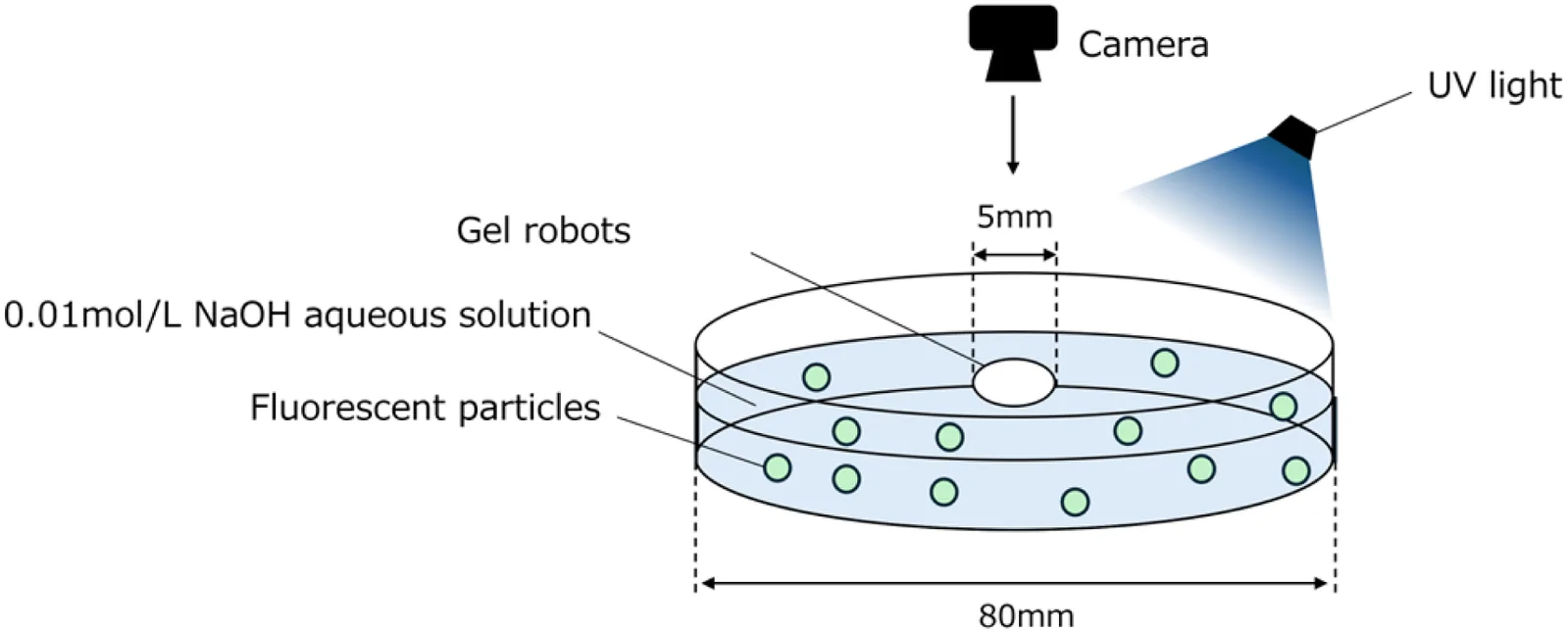
Schematic illustration of the experimental setup.

A 0.01 mol per L NaOH aqueous solution containing a small amount of dispersed fluorescent particles for convection visualization is prepared, and 30 mL of the solution is poured into a Petri dish. Subsequently, each robot, cut into a 5-mm-diameter disk, is immersed in the solution. Under UV light irradiation (TA434KA, Nichia Corporation), the motion of the gel robots and the corresponding convection patterns are recorded using a video camera (EOS 80D, Canon Inc.). The experimental setup is illustrated in Fig. 6. Finally, the self-propelled behavior is analyzed using the video analysis software Kinovea. The above procedure is repeated five times for each robot.

### 4.2 Directional control of the gel robots

This section proposes a directional control method that introduces an exoskeleton frame. The objective is to provide directionality by physically shielding the perimeter of the 1-pentanol-containing gel robot and geometrically restricting the reaction interface.

A gel robot equipped with an exoskeleton frame reacts with the external solution only at specific locations, thereby limiting the release points of the droplets. As a result, the formation of convection associated with the reaction is locally restricted, thus directionality is imparted to the self-propulsion behavior.

The exoskeleton frame is designed in a bow (half-moon) shape, with an opening located at the center of the chord. This design ensures that the propulsion vector generated by external convection acts along the central axis of the gel, which is expected to initiate self-propulsion in the direction of the bow. The frame is designed using Autodesk Fusion, and the slicing data is generated using Snapmaker Luban. Subsequently, the frame is fabricated using a fused deposition modeling (FDM) 3D printer.

**Fig. 7:**
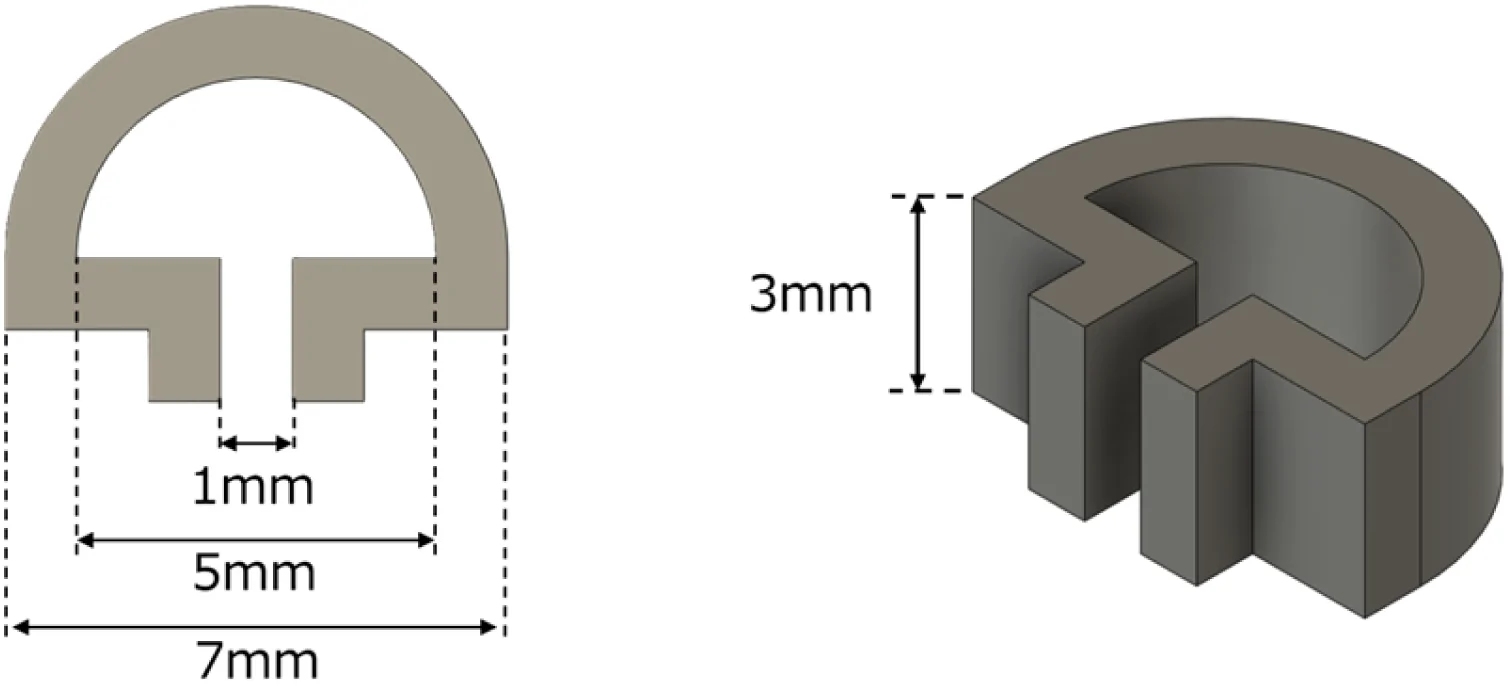
Design of the exoskeleton frame.

The employed hardware is a Snapmaker A035T equipped with a 3D Printing Module Dual Extrusion, and PLA (polylactic acid) is used as the printing material. The design diagram of the fabricated exoskeleton frame is shown in Fig. 7.

To evaluate the self-propulsion and convection patterns, we first prepare a 0.01 mol per L NaOH aqueous solution with dispersed fluorescent particles. We then mold the porous organogel and the 1-pentanol solvent-based organogel into the shape of the fabricated exoskeleton frame and attach them to the frame.

Before the observation experiment, we wash the frame-equipped gel with PBS and carefully wipe off any surface moisture. The sample is then positioned at the center of a Petri dish with a diameter of 8 cm, which is filled with 30 mL of the prepared NaOH aqueous solution. We record the behavior of the gel robot for 30 minutes using a video camera under UV light irradiation. Finally, the self-propulsion behavior of the gel is analyzed using the video analysis software, Kinovea.

Furthermore, convection analysis is conducted using Particle Image Velocimetry (PIV) to visualize the external convection formed in the solution when the exoskeleton frame is attached. In this system, a new fixation frame was fabricated using a 3D printer to secure the porous organogel robot near the center of the Petri dish for imaging.

**Fig. 8:**
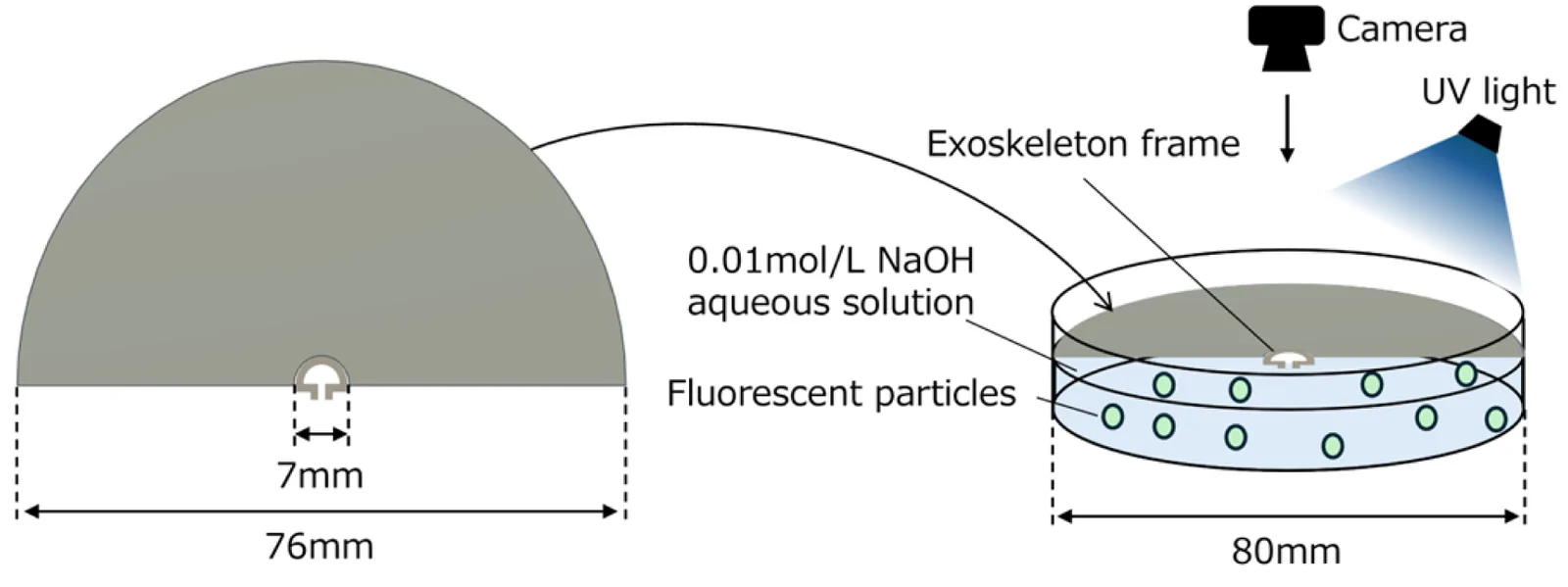
Schematic figures of the experimental setup for convection analysis.

A schematic of the experimental setup is shown in Fig. 8. A high-speed camera (Baumer VUXU-13M) is employed for image acquisition.

## 5 Results and discussion

### 5.1 Evaluation of self-propelled behavior

As a result of the experiment using the emulsion gel robot, the self-propulsion of the hydrogel was observed, driven by the release of 1-pentanol droplets over time.

**Fig. 9:**
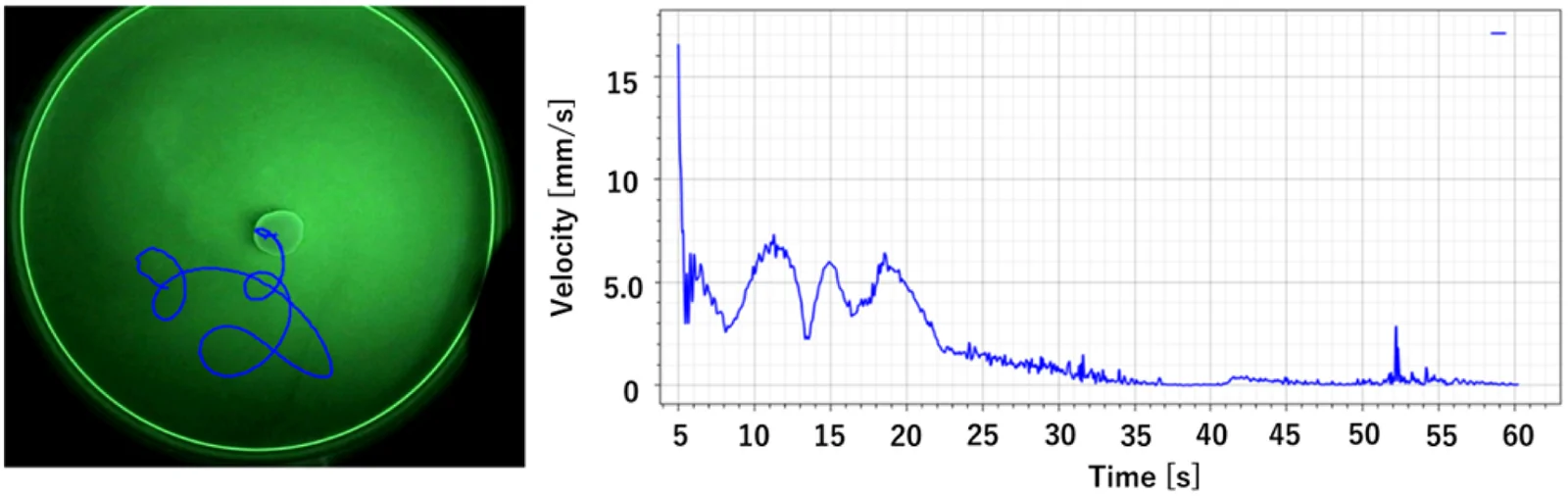
Self-propulsion trajectory and velocity changes of the emulsion gel robot for approximately 1 minute immediately after immersion.

**Fig. 10:**
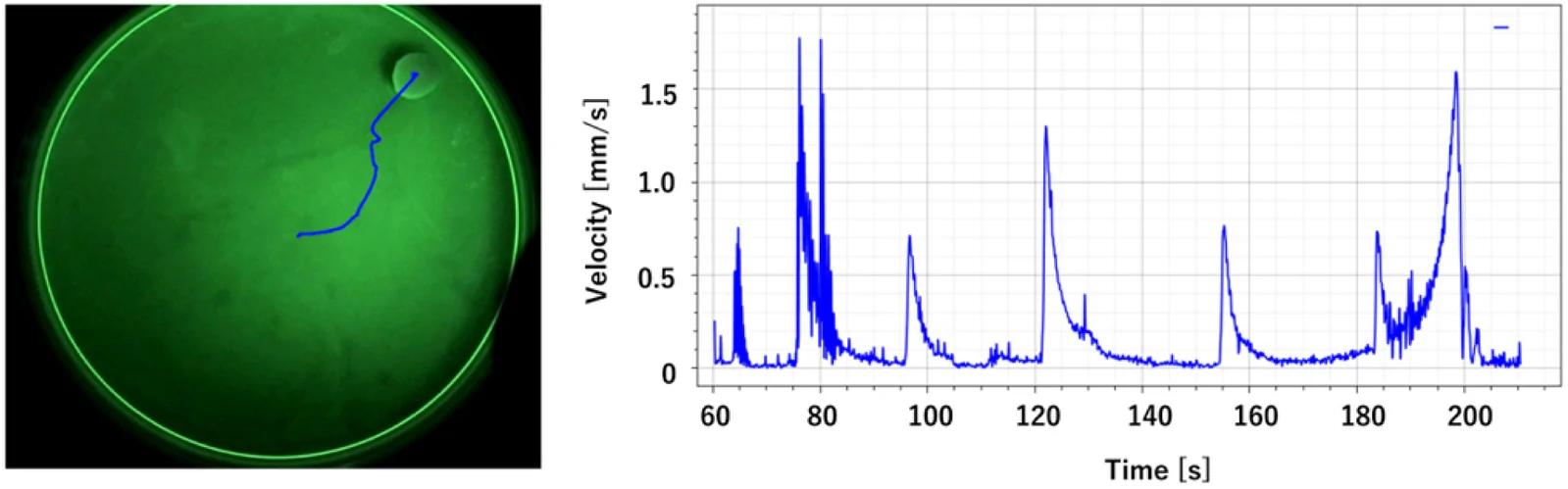
Self-propulsion trajectory and velocity changes of the emulsion gel robot for approximately 150 seconds after stabilization.

As a representative example from five trials, Fig. 9 shows the trajectory and velocity of the self-propulsion for approximately one minute immediately after immersion in the NaOH aqueous solution. Additionally, Fig. 10 shows the trajectory and velocity for the subsequent 150 seconds. The behaviors of the self-propulsion are shown in Movies S1 and S2.

As shown in Fig. 9, the gel began self-propelling immediately after immersion and then transitioned into a quiescent state with almost no velocity after a certain period. Subsequently, as seen in Fig. 10, the gel resumed self-propulsion; however, this motion exhibits pulsatile behavior, characterized by alternating periods of stop and propulsion.

Regarding the behavior immediately after immersion, the initial continuous self-propulsion is likely attributed to the consumption of 1-pentanol present near the gel surface. As the concentration of 1-pentanol near the surface decreased after a period of motion, the self-propulsion ceased.

Two primary factors are considered to explain the subsequent quiescent state. First is the swelling speed associated with the pH-responsiveness of the hydrogel. Under basic conditions, the polymer network expands due to electrostatic repulsion caused by the dissociation of carboxyl groups. It is thought that a certain amount of time was required for the network to expand sufficiently relative to the droplet size to allow for the release of the encapsulated 1-pentanol. Second is the diffusion time of the 1-pentanol droplets. The droplets dispersed within the hydrogel must move toward the surface *via* diffusion to be released.

The pulsatile behavior observed in Fig. 10 can also be explained by the diffusion time of the droplets. The external convection that serves as the propulsive force depends on the concentration gradient of 1-pentanol at the hydrogel surface. Once convection is formed and the gel propels, the droplets at the surface are consumed, leading to a temporary decrease in 1-pentanol concentration. To form the convection again, the dispersed droplets inside the gel must reach the surface to form a sufficient concentration gradient, which requires a specific time interval. Consequently, this inherent time lag between the rapid consumption of surface droplets and their replenishment from the interior explains why the hydrogels exhibit a recurring cycle of rest and propulsion. Although the magnitude of the velocity peaks varies quantitatively in each event, this fundamental cycle driven by droplet diffusion remains consistent.

The experimental results for the porous organogel robot showed that, like the emulsion hydrogel, the gel began self-propelling immediately after immersion in the solution. After a certain period, it exhibited a motion pattern characterized by pulsatile movement, alternating between stop and propulsion.

**Fig. 11:**
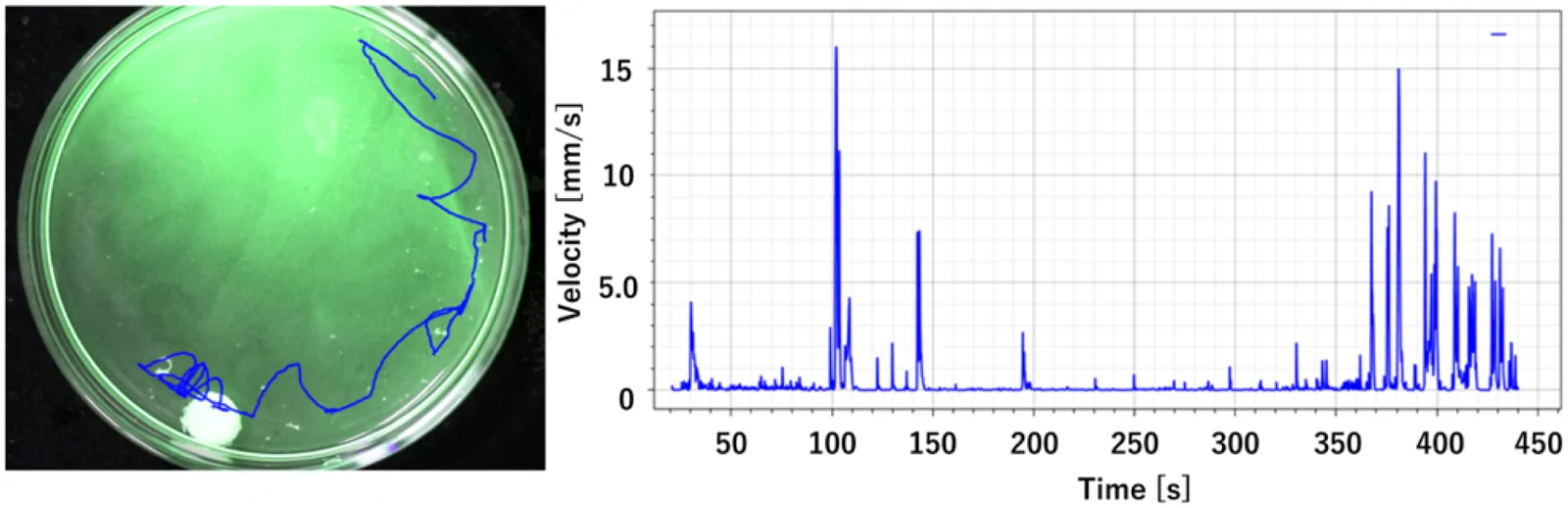
Self-propulsion trajectory and velocity changes of the porous organogel robot for approximately 7 minutes.

As a representative example from five trials, Fig. 11 shows the trajectory over approximately 7 minutes after the self-propulsion stabilized, along with a graph representing the changes in velocity during motion. The behavior of the self-propulsion is shown in Movie S3.

Furthermore, a significant improvement in the duration of self-propulsion was observed. This is attributed to the unique internal structure of the porous organogel.

The porous organogel possesses a network of pores formed during the drying process, and re-swelling causes 1-pentanol to fill the pore channels.

Consequently, these pores are considered to function not only as reservoirs for the droplets but also as transport channels from the gel's inside to its surface.

As a result, when droplets on the surface are consumed, they are rapidly replenished from the inside through the pore network, thereby sustaining self-propulsion over an extended period.

**Fig. 12:**
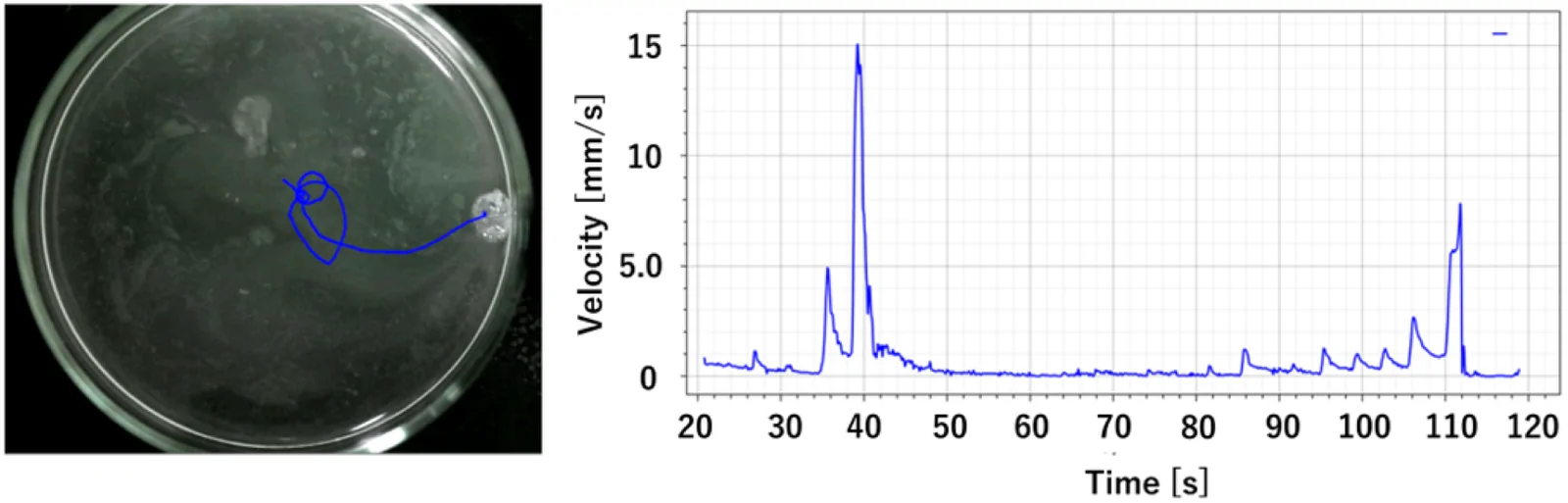
Self-propulsion trajectory and velocity changes of the 1-pentanol solvent-based organogel robot for approximately 100 seconds.

The experimental results for the 1-pentanol solvent-based organogel robot showed that, similar to the emulsion hydrogel and the porous organogel, the gel began self-propelling immediately after immersion in the solution. After a certain period, it exhibited a motion pattern of pulsatile propulsion, alternating between stop and movement. As a representative example from five trials, Fig. 12 shows the trajectory for approximately 100 seconds after the self-propulsion stabilized, along with a graph representing the changes in velocity during motion. The self-propulsion behavior is shown in Movie S4.

Consequently, a decrease in the duration of self-propulsion was observed compared to the porous organogel robot fabricated through the drying and re-swelling process. The following discussion focuses on the internal structure of the gel to explain this result.

It was inferred that the porous organogel maintained long-term self-propulsion by securing release pathways for droplets through its porous structure.

In contrast, the 1-pentanol solvent-based organogel has a structure where 1-pentanol is uniformly dispersed within the gel, and no clear release pathways exist. Therefore, internal droplets must move through the dense network *via* diffusion to be released. As a result, when droplets on the gel surface are consumed, the supply from the inside cannot keep pace; thereby, self-propulsion ceases.

**Fig. 13:**
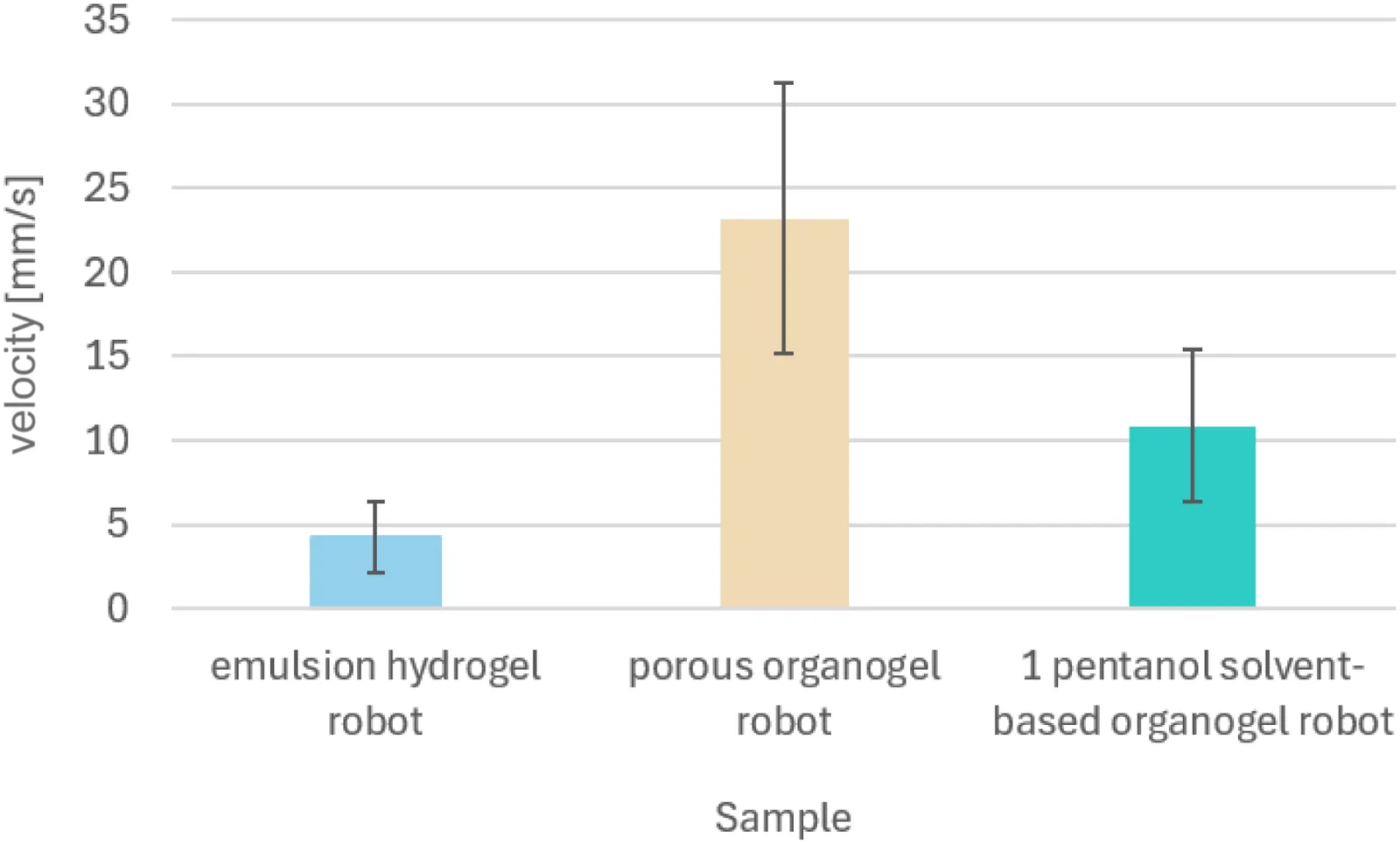
Mean maximum velocity and standard deviation for each gel robot.

The mean maximum velocity and standard deviation were calculated for each of the three types of robots based on the results of the five trials. The calculated results are presented in Fig. 13.

The mean maximum velocity of the three gel robots increased in the order of the emulsion hydrogel robot, the 1-pentanol solvent-based organogel robot, and the porous organogel robot. This trend is consistent with the maximum velocity results observed for each robot under the pH 12 environment, as discussed in Section 3.2.

The results comparing the fabrication efficiency, physical properties, and self-propulsion characteristics for each method are summarized in Table 1.

**Table 1:** Comparison of Fabrication Methods

Method	Polymerization time	Structural stability	Droplet retention capacity	Mean maximum velocity [mm s^−1^] (pH 12)
Emulsion hydrogel robot	24 hours	Phase separation	Dependent on emulsion stability	4.26
Porous organogel robot	30 minutes (drying and reswelling: 24 hours)	Uniform polymerization	Stable packing	23.2
1-Pentanol solvent-based organogel robot	30 minutes	Uniform polymerization	Stable dispersion	10.8

As shown in Table 1, the porous organogel fabricated by the drying and re-swelling process demonstrates high performance across all evaluation criteria, except for fabrication time. In contrast, the 1-pentanol solvent-based organogel shows superior performance in all categories except for the mean maximum velocity.

Based on these results, it is concluded that the porous organogel is the most effective fabrication method when self-propulsion maximum velocity is prioritized. In contrast, the 1-pentanol solvent-based organogel is most suitable when the primary focus is on fabrication efficiency.

Pulsatile self-propulsion behavior, characterized by alternating cycles of stop and movement, was consistently observed across all three fabrication methods.

This phenomenon is attributed to the swelling rate associated with the pH-responsiveness of the PNIPAm-*co*-AAc hydrogel and the diffusion time of the 1-pentanol droplets. Consequently, the gel robots developed in this study have been established as a system capable of autonomous motion in response to environmental pH changes.

### 5.2 Directional control of the gel robots

**Fig. 14:**
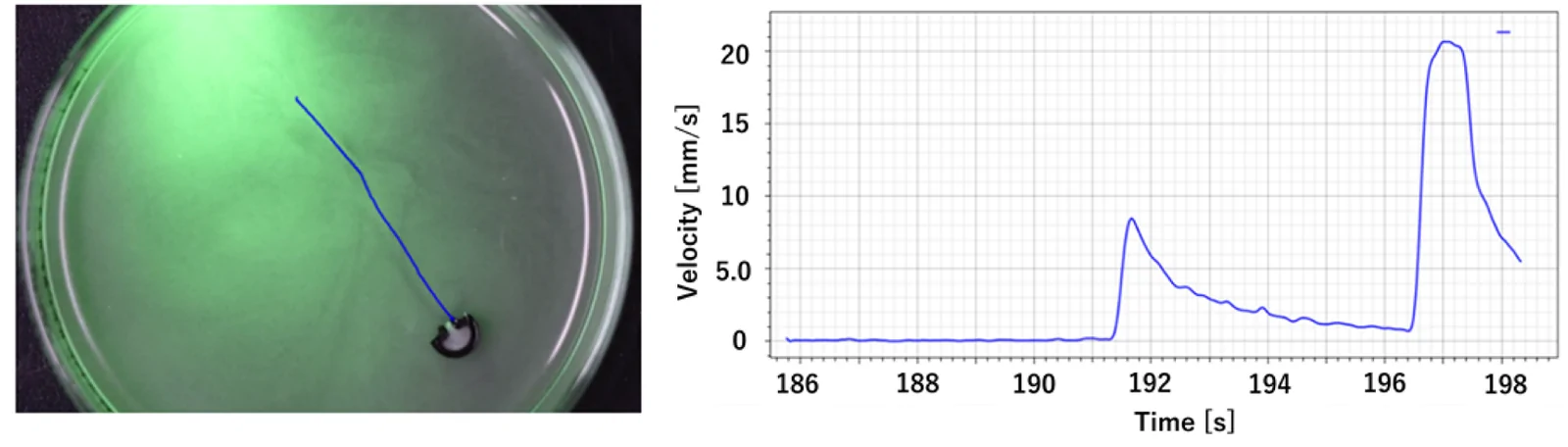
Straight trajectory and velocity changes of the porous organogel robot.

Fig. 14 shows the straight trajectory and velocity changes of the porous organogel robot.

**Fig. 15:**
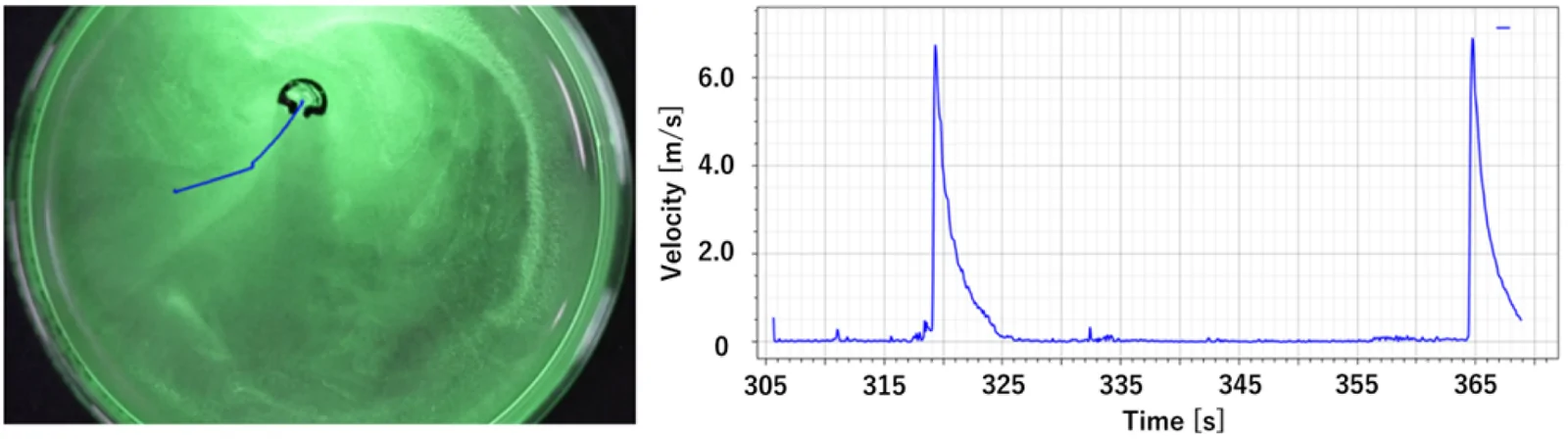
Straight trajectory and velocity changes of the 1-pentanol solvent-based organogel.

As indicated in Fig. 14, the robot exhibited two distinct propulsion phases as droplets were released over time, and it can be confirmed that a stable straight trajectory was achieved. Similarly, Fig. 15 below shows the straight trajectory and velocity changes for the 1-pentanol solvent-based organogel robot obtained under the same experimental conditions.

Fig. 15 shows that a straight trajectory is maintained across the two propulsion phases. However, an increase in the duration of the stationary phase is observed compared to the experiment using the porous organogel (Fig. 14). The self-propulsion behaviors shown in Fig. 14 and 15 are demonstrated in Movies S5 and S6.

Due to this extended stationary phase, the robot is subjected to the influence of the surrounding convection induced by the initial propulsion for a longer period, resulting in subtle angular changes during the period of rest. Consequently, a slight mismatch occurs between the first and second propulsion vectors; thereby, the trajectory deviates from a perfectly linear path.

In the comparison of the self-propulsion experiments in the previous experiments (Fig. 14 and 15), a significant increase in the duration of the stationary phase was observed as a major difference. Based on the discussion in Section 5.1, this phenomenon is inferred to be influenced by the diffusion rate of 1-pentanol. Therefore, it is considered that increasing the solution temperature within a range not exceeding the LCST will promote internal diffusion; thereby, the stationary phase can be shortened.

**Fig. 16:**
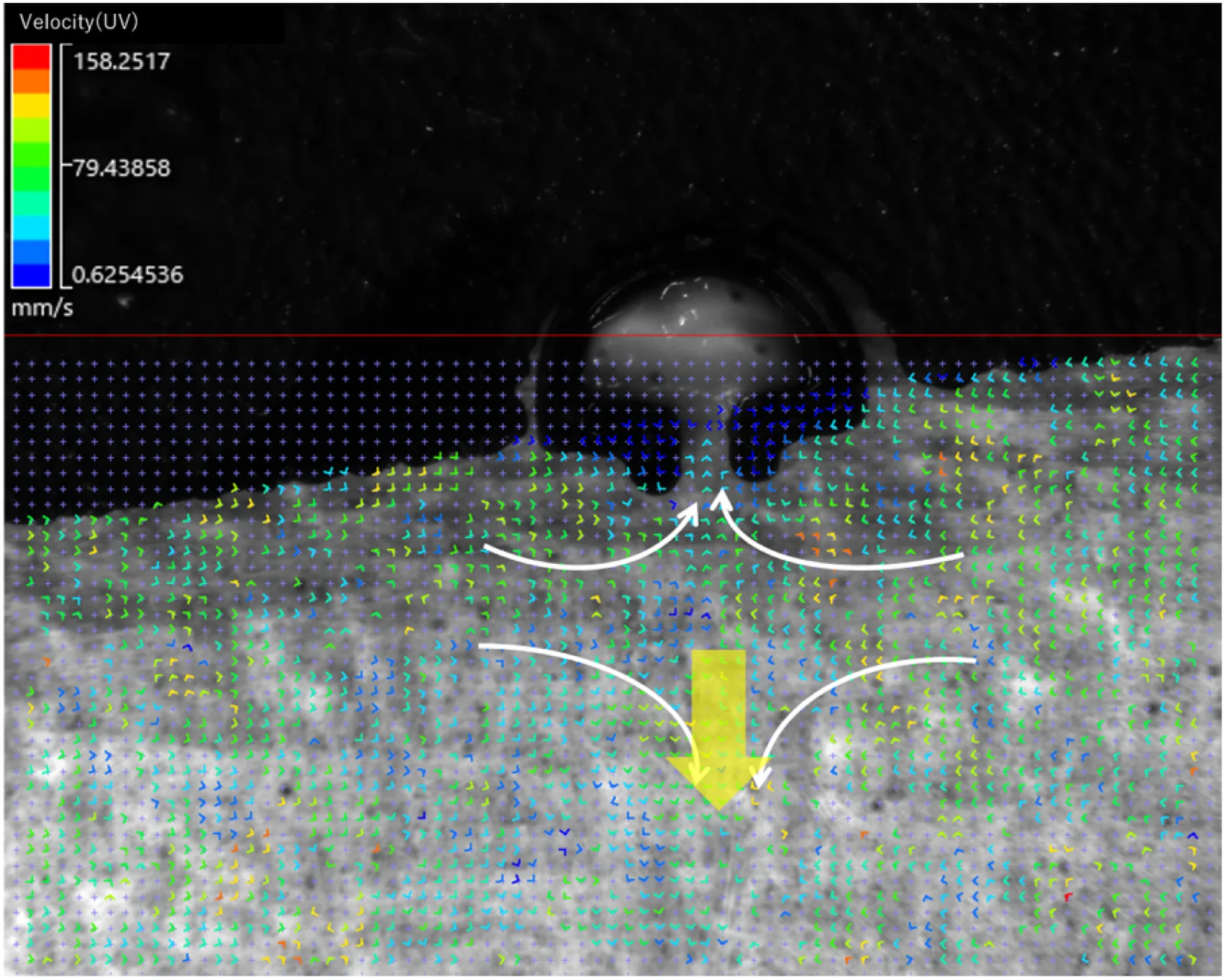
Results of convection analysis using the porous organogel robot.

The results of the convection analysis obtained from the experiments are shown in Fig. 16. As shown in Fig. 16, convection moving outward from the vicinity of the frame opening and being drawn into the opening are observed. Additionally, it can be confirmed that the outward convection, which serves as the propulsion force, is aligned with the central axis of the gel. The convection drawn into the vicinity of the frame opening results from an inflow of the surrounding solution to compensate for the fluid volume displaced *via* diffusion.

In summary, the introduction of the exoskeleton frame enables stable control of the straight trajectory. This precise motion control is confirmed through the combined evidence of the trajectory and velocity results, as well as the convection analysis near the opening.

## 6 Conclusions

In this study, we developed a composite system combining 1-pentanol, a type of self-propelling droplet, with PNIPAm-*co*-AAc hydrogel and investigated the controllability of its self-propulsion behavior in response to pH changes. The findings obtained through this research are summarized below.

First, regarding the construction of gel robots containing 1-pentanol, three different fabrication methods were compared and evaluated.

### 6.1 Emulsion hydrogel robot

Clear self-propulsion behavior was confirmed in basic solutions.

### 6.2 Porous organogel robot

A significant extension of self-propulsion duration was observed compared to the emulsion hydrogel robot. This is attributed to the internal porous structure functioning as both a reservoir and a diffusion pathway for the droplets; thereby, an efficient supply of droplets to the gel surface is enabled.

### 6.3 1-Pentanol solvent-based organogel robot

While this method required the shortest fabrication time among the three methods, a decrease in self-propulsion duration was observed compared to the porous organogel robot.

In all three fabrication methods, pulsatile self-propulsion, characterized by alternating rest and movement, was observed. This phenomenon is attributed to the diffusion time required for droplets to migrate from the interior to the surface in response to pH changes.

Second, regarding the controllability of the self-propulsion behavior, we successfully imparted a specific directionality to the robot—which previously exhibited random motion—by using 3D-printed exoskeleton frames to geometrically restrict the reaction interface. Furthermore, based on convection analysis using Particle Image Velocimetry (PIV), we confirmed the convection generated in the vicinity of the porous organogel robot.

In conclusion, through this research, we have established guidelines for constructing environment-responsive self-propelling robots that drive autonomously in basic solutions by utilizing the pH-responsiveness of PNIPAm-*co*-AAc hydrogels.

Although this study established the construction guidelines for 1-pentanol-containing gel robots, complex control over their self-propulsion behavior has yet to be fully realized. Therefore, future work will aim to develop more dynamic self-propulsion systems with the objective of advancing environmental responsiveness and enhancing directional controllability.

## Author contributions

Ittetsu Toda performed all experiments and wrote the manuscript. Hideyuki Sawada supervised the research project and wrote the manuscript. All authors discussed the results.

## Conflicts of interest

There are no conflicts to declare.

## Supplementary Material

RA-OLF-D6RA03989H-s001

RA-OLF-D6RA03989H-s002

RA-OLF-D6RA03989H-s003

RA-OLF-D6RA03989H-s004

RA-OLF-D6RA03989H-s005

RA-OLF-D6RA03989H-s006

RA-OLF-D6RA03989H-s007

## Data Availability

The data supporting this article have been included as part of the supplementary information (SI). Supplementary information is available. See DOI: https://doi.org/10.1039/d6ra03989h.
