# Supplementary material for: Development of environment-responsive self-propelling robots with shape-preserving exoskeletons for directed interfacial motion
Source: RSC Adv. 2026 Aug 25;16(42):50461–9. doi: 10.1039/d6ra03989h (PMC13505774; doi:10.1039/d6ra03989h)
Supplement: Supplementary file 1 [file RA-016-D6RA03989H-s001.pdf]

Supplementary Information (SI) for RSC Advances.
This journal is © The Royal Society of Chemistry 2026

Supplementary Information

Development of Environmentally Responsive Self-Propelling Robots in Solution Using PNIPAm-co-AAc Hydrogels

Ittetsu Toda*[a] and Hideyuki Sawada[b]

[a] Department of Pure and Applied Physics, Graduate School of Advanced Science and Engineering, Waseda University, 3-4-1 Okubo, Shinjuku-Ku, Tokyo, 169-8555, Japan.

[b] Faculty of Science and Engineering, Waseda University, 3-4-1 Okubo, Shinjuku-Ku, Tokyo, 169-8555, Japan.

Contents

S1. Supporting video information 2

S1. Supporting video information

Movie S1: Self-propulsion behavior of the emulsion gel robot for approximately 1 minute immediately after immersion (5× speed).

Movie S2: Self-propulsion behavior of the emulsion gel robot for approximately 150 seconds after stabilization (5× speed).

Movie S3: Self-propulsion behavior of the porous organogel robot for approximately 7 minutes (10× speed).

Movie S4: Self-propulsion behavior of the 1-pentanol solvent-based organogel robot for approximately 100 seconds (5× speed).

Movie S5: Self-propulsion behavior of the porous organogel robot with an exoskeleton. (1× speed).

Movie S6: Self-propulsion behavior of the 1-pentanol solvent-based organogel with an exoskeleton. (5× speed).
